# Streptococcal Receptor Polysaccharides: Recognition Molecules for Oral Biofilm Formation

**DOI:** 10.1186/1472-6831-6-S1-S12

**Published:** 2006-06-15

**Authors:** Yasuo Yoshida, Robert J Palmer, Jinghua Yang, Paul E Kolenbrander, John O Cisar

**Affiliations:** 1 Oral Infection and Immunity Branch, National Institute of Dental and Craniofacial Research, National Institutes of Health, Bethesda, MD 20892-4352, USA

## Abstract

**Background:**

Strains of viridans group *streptococci *that initiate colonization of the human tooth surface typically coaggregate with each other and with *Actinomyces naeslundii*, another member of the developing biofilm community. These interactions generally involve adhesin-mediated recognition of streptococcal receptor polysaccharides (RPS). The objective of our studies is to understand the role of these polysaccharides in oral biofilm development.

**Methods:**

Different structural types of RPS have been characterized by their reactions with specific antibodies and lectin-like adhesins. Streptococcal gene clusters for RPS biosynthesis were identified, sequenced, characterized and compared. RPS-producing bacteria were detected in biofilm samples using specific antibodies and gene probes.

**Results:**

Six different types of RPS have been identified from representative viridans group *streptococci *that coaggregate with *A. naeslundii*. Each type is composed of a different hexa- or heptasaccharide repeating unit, the structures of which contain host-like motifs, either GalNAcβ1-3Gal or Galβ1-3GalNAc. These motifs account for RPS-mediated recognition, whereas other features of these polysaccharides are more closely associated with RPS antigenicity. The RPS-dependent interaction of *S. oralis *with *A. naeslundii *promotes growth of these bacteria and biofilm formation in flowing saliva. Type specific differences in RPS production have been noted among the resident streptococcal floras of different individuals, raising the possibility of RPS-based differences in the composition of oral biofilm communities.

**Conclusion:**

The structural, functional and molecular properties of streptococcal RPS support a recognition role of these cell surface molecules in oral biofilm formation.

## Background

Colonization of the human tooth surface is initiated by a limited number of gram-positive species, primarily viridans group *streptococci *[1]. These bacteria attach to host salivary components that coat the mineral surface and, through growth and interactions between species, form a relatively simple biofilm community (i.e. early plaque). Members of this community can activate host cells, and the biofilm itself creates a habitat for additional species, some of which are closely associated with the initiation and progression of dental caries and periodontal disease [2]. In the current communication we summarize evidence for the hypothesis that the surface polysaccharides of certain viridans group streptococci are receptors for lectin-like adhesins present on other members of the developing oral biofilm community. Studies of how these components influence biofilm development may provide insights into the etiology and pathogenesis of biofilm-related oral diseases.

### Early Biofilm Formation Involves a Limited Number of Microbial Species

An attractive experimental model for defining early events in oral biofilm formation involves the incubation of retrievable enamel chips in the human oral cavity for periods of time from 4 to 12 hrs. Scanning electron micrographs of chips removed during this period suggest that colonization is initiated by the attachment of individual microorganisms to the saliva-coated mineral surface and proceeds primarily by the growth of attached bacteria as spreading microcolonies [3]. These microcolonies typically consist of a peripheral monolayer of dividing cocci and rods and a central region containing multiple bacterial layers. *S. sanguinis*, *S. oralis *and *S. mitis *were the most prominent species identified in parallel microbiological studies [2] and together comprised approximately 80% of the total cultivatable flora. Gram-positive pleomophic rods, mostly strains of *Actinomyces naeslundii*, made up another 5 to 10% of the total. Thus, primary colonization of the saliva-coated mineral surface involves the formation of a relatively simple biofilm community.

Interestingly, *S. mutans*, the principal agent of human dental caries, was generally not identified from early biofilms formed on enamel chips following incubation in the oral cavities of individuals with active caries [4]. Moreover, when this species was detected, it accounted for less than 2% of the total early streptococcal flora. Differences in the relative proportions of other streptococcal species were however noted between the early flora of caries active and caries free individuals. Specifically, the proportion of *S. mitis *was generally elevated at the expense of *S. sanguinis* in caries active individuals. The ecological consequences of this population shift are not well understood. However, they may include a lowering of the early plaque pH as suggested from comparative studies of acid production by representative strains of these streptococcal species [5]. A more acidic environment could in turn favor the succession of aciduric species such as *S. mutans*, resulting in rapid caries development. Thus, the etiology and pathogenesis of oral disease may well be linked to ecological properties of the early biofilm community.

### Structural Basis of Receptor Polysaccharide (RPS)-Mediated Interbacterial Adhesion

Bacterial properties that contribute to development of the early biofilm community may include the surface adhesins and receptors that mediate the coaggregations observed between different strains of *S. sanguinis*, *S. gordonii*, *S. oralis*, *S. mitis *and *A. naeslundii *[6]. The coaggregations between *A. naeslundii *and different *streptococci *are typically inhibited by GalNAc or Gal and result from lectin-like binding of *A. naeslundii *type 2 fimbriae to complementary receptors present on the streptococcal surface. These receptors occur on most strains of *S. oralis *and on certain strains of *S. sanguinis*, *S. gordonii *and *S. mitis*. Many but not all *streptococci *that possess such receptors also participate in coaggregations with other strains of *S. sanguinis* or *S. gordonii*. These coaggregations are inhibited by GalNAc and result from lectin-like binding of surface adhesins present on the strains of *S. sanguinis* or *S. gordonii *[7]. Thus, an extensive network of adhesive interactions occurs among different members of the early biofilm community.

The structural basis of the coaggregations observed between different bacteria is evident from the carbohydrate-binding specificities of the surface adhesins present on strains of *A. naeslundii*, *S. sanguinis* and *S. gordonii*, and from the structures of the complementary receptors present on other streptococcal strains. The type 2 fimbriae of *A. naeslundii*, in addition to mediating coaggregations with *streptococci*, mediate the adhesion of *A. naeslundii *to surface-associated host glycoconjugates [8,9]. Two different receptor structures have been identified, terminal GalNAcβ1-3Gal of globoside and terminal Galβ1-3GalNAc of asialo-*O*-linked glycoproteins and gangliosides. Consistent with these findings, soluble GalNAcβ1-3Gal- and Galβ1-3GalNAc-containing ligands are potent inhibitors of *A. naeslundii *type 2 fimbriae-mediated adhesion [10]. These disaccharides differ only in the position of the N-acetyl group. Consequently, the ability of each to inhibit binding of *A. naeslundii *type 2 fimbriae presumably depends on common features of these structures. The GalNAc-binding surface adhesins of *S. sanguinis*SK1 and *S. gordonii *DL1 also recognize terminal GalNAcβ1-3Gal [11]. However, these adhesins do not bind terminal Galβ1-3GalNAc of immobilized glycocongugates. In accordance with these findings, the coaggregations observed between strains of *S. sanguinis* or *S. gordonii *and other *streptococci *are inhibited more effectively by soluble GalNAcβ1-3Gal- than Galβ1-3GalNAc-containing ligands [6]. Thus, the N-acetyl group of GalNAcβ1-3Gal is critical for binding of the streptococcal adhesins that mediate these interactions.

**Figure 1:**
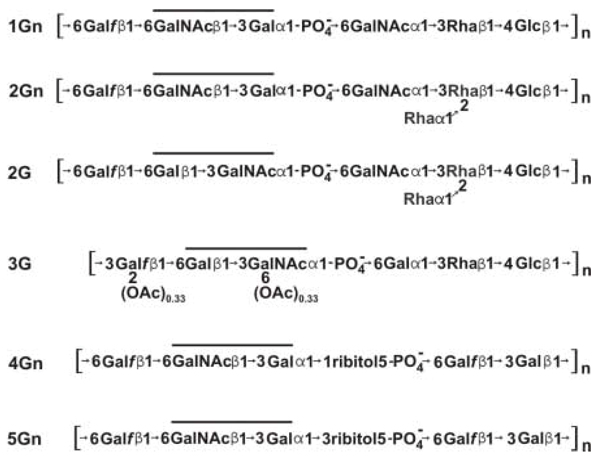
The six structural types of RPS identified from strains of *S. sanguinis*, *S. gordonii*, *S. oralis *and *S. mitis *that coaggregate with *A. naeslundii*. Partial O-acetylation of type 3G RPS is indicated. Lines indicate the location of GalNAcβ1-3Gal (Gn) or Galβ1-3GalNAc (G) recognition motifs within the hexa- or heptasaccharide repeating units of different RPS structural types.

Structural studies of over 20 representative strains of *S. sanguinis*, *S. gordonii*, *S. oralis *and *S. mitis *that participate in type 2 fimbriae-mediated coaggregations with *A. naeslundii *have resulted in the identification of six different streptococcal receptor polysaccharides (RPS), each composed of a distinct hexa- or heptasaccharide repeating unit (Fig. 1) [12]. Remarkably, a host-like receptor motif for binding of *A. naeslundii *type 2 fimbriae occurs within each RPS repeating unit. Four structural types of RPS contain GalNAcβ1-3Gal (Gn) motifs (i.e., RPS types 1Gn, 2Gn, 4Gn and 5Gn) while the other two types contain Galβ1-3GalNAc (G) motifs (i.e., RPS types 2G and 3G). All streptococci that bear these polysaccharides are coaggregation partners of *A. naeslundii*. However, only streptococci that bear Gn-types of RPS are coaggregation partners of *S. sanguinis*SK1 and *S. gordonii *DL1. The GalNAc binding adhesins of these strains do not recognize G-types of RPS. Thus, the occurrence of Gn and G types of RPS on different streptococci may influence biofilm development.

Other features of RPS that are conserved between structural types may contribute to adhesin binding of these linear polysaccharides. Such features include the flexibility of the β1–6 linkage from Gal*f *and the anionic phosphodiester bond that flank each recognition motif [13,14]. This region, although critical for interbacterial adhesion, contributes little to the antigenicity of different RPS structural types. Instead, other structural features such as the Rha branch present in types 2Gn and 2G RPS [15] form the major epitopes of anti-RPS antibodies. Consequently, the reactions of types 2Gn and 2G RPS are similar as antigens but distinct as receptors. Other structural types such as types 1Gn and 2Gn RPS react as similar receptors but different antigens. Such findings raise the possibility that the production of different antigenic types of RPS contributes to evasion of the host secretory immune response during oral biofilm formation. However, the presence of specific anti-RPS antibodies has not been detected in samples of whole human saliva. Thus, further studies of the host response (or the lack of a response) to these molecules are needed.

### Molecular Basis of RPS Structure and Function

**Figure 2:**
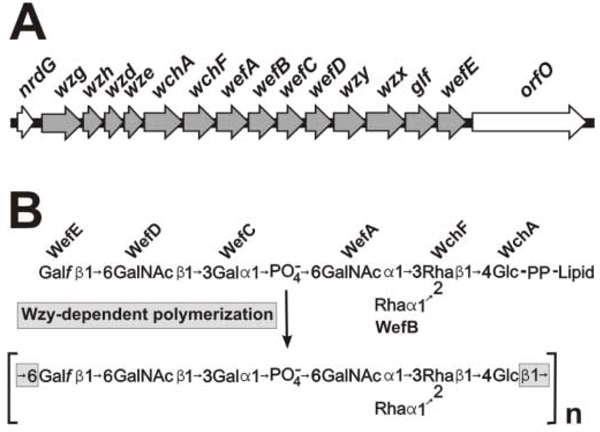
Molecular basis of type 2Gn RPS biosynthesis and structure. (A) The operon for type 2Gn RPS biosynthesis in *S. gordonii *38 consists of 14 genes located between *nrdG *and *orfO*. The first four of these encode regulatory proteins. The remaining 10 genes include seven for putative glycosyltransferases, *wzx *and *wzy *for the transport and polymerization of RPS repeating units and *glf *for the epimerase that supplies UDP-Gal*f*, one of five essential RPS precursors. (B) The deduced roles of the seven glycosylytransferases encoded by the type 2Gn RPS gene cluster in synthesis of the corresponding lipid-linked heptasaccharide repeating unit. The β1–6 linkage between Glc and Gal*f *in type 2Gn RPS is formed by Wzy-dependent polymerization of RPS repeating units.

Results from recent molecular studies have identified biosynthetic gene clusters for each of the six RPS structural types. As illustrated for the type 2Gn RPS gene cluster of *S. gordonii *38 (Fig. 2) [16], the 5'-ends of each RPS cluster contain four common regulatory genes (*i.e*., *wzg*, *wzh*, *wzd *and *wze*). Homologues of these genes occur widely in polysaccharide gene clusters of gram-positive bacteria, including capsular polysaccharide genes clusters of *S. pneumoniae *[17]. The regulatory region of each RPS gene cluster is followed by genes for the glycosyltransferases that synthesize the lipid-linked, type-specific RPS repeating unit. High homology exists between a number of genes for glycoslytransferases in strains of *S. gordonii *and *S. oralis *[18], suggesting horizontal gene transfer between these species. Moreover, the presence or absence of specific genes is closely correlated with structural differences that exist between different types of RPS. For example, the Rha branch that distinguishes type 2Gn from 1Gn RPS depends on the glycosyltransferase encoded by *wefB*, a gene that is present in the type 2Gn RPS cluster of *S. gordonii *38 but not in the type 1Gn RPS gene cluster of *S. oralis *34 [18]. Likewise, different pairs of adjacent genes, *wefC *and *wefD *in the type 2Gn cluster of *S. gordonii *38 and *wefF *and *wefG *in the type 2G cluster of *S. oralis *J22, account for the distinct recognition motifs present in the corresponding polysaccharides of these strains [19]. Additional genes in each RPS cluster encode a putative flipase (Wzx), which transports the oligosaccharide moiety of the lipid-linked repeating unit to the outer surface of the cytoplasmic membrane and a putative polymerase (Wzy), which links repeating units end-to-end forming the linear polysaccharide chain. Polymerization involves the formation of a glycosidic linkage between the first and last sugars of each RPS repeat (*i.e*., Glcβ1-6Gal*f *in types 1Gn, 2Gn and 2G RPS, Glcβ1-3Gal*f *in type 3G RPS and Galβ1-6Gal*f *in types 4Gn and 5Gn RPS).

Additional genes control the synthesis of nucleotide-linked sugars that are essential precursors for RPS biosynthesis. One of these (*glf*) encodes galactofuranose mutase, the enzyme that catalyzes the conversion of UDP-Galactopyranose to UDP-Galactofuranose. This gene invariably occurs within the RPS gene clusters of different strains. The location of other genes for nucleotide sugar biosynthesis varies between streptococcal species. For example, in *S. gordonii *38, the first three genes of the dTDP-**L**-Rhamnose biosynthetic pathway (*i.e*., *rmlA*, *rmlC *and *rmlB*) occur in a separate operon with *galE2*, the gene for a bifunctional galactose epimerase that supplies both UDP-Gal and UDP-GalNAc for polysaccharide biosynthesis [16]. The final gene for dTDP-**L**-Rhamnose biosynthesis (*i.e., rmlD*) is transcribed independently, downstream of the *rml*-*galE2 *operon. In contrast, *rmlA*, *rmlC *and *rmlB *of *S. oralis *strains 34 and J22 occur at the 3'-end of the respective type 1Gn and 2G RPS gene clusters of these strains, while *rmlD *occurs immediately downstream but transcribed in the opposite direction [19,18]. The differences that exist between the RPS gene clusters of *S. gordonii *and *S. oralis*, both in terms of their organization and nucleotide sequence homologies, raise the interesting possibility that the evolution of RPS production in these bacteria occurred following the divergence of these species. The four RPS-producing species of oral streptococci, *S. sanguinis*, *S. gordonii*, *S. oralis *and *S. mitis*, are closely related members of the mitis group [20]. Members of this group, which includes *S. pneumoniae*, are all intimately associated with the oral cavity and upper respiratory tract of man. Thus, the evolution of certain species as members of the human oral biofilm community may be closely linked with the evolution of RPS production. The identification of genes for RPS structure and function provides a beginning basis for further phylogenetic studies to examine this intriguing possibility.

### Role of RPS-mediated Interbacterial Adhesion in Biofilm Development

**Figure 3:**
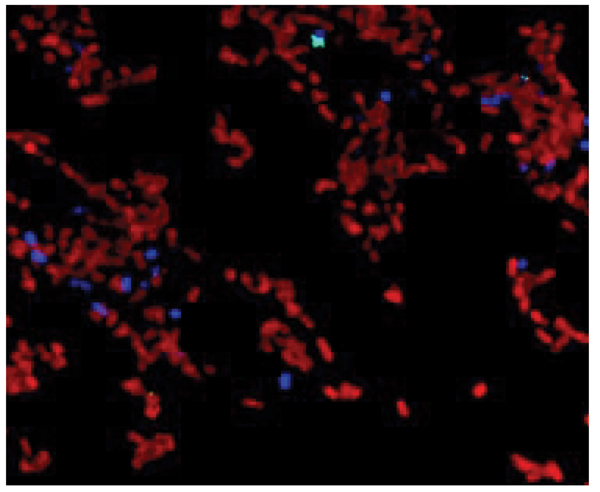
Laser confocal micrograph of an early biofilm formed on the surface of an enamel chip incubated 4 hr in vivo showing the presence of immono-labeled *A. naeslundii *(blue) within the monolayer of immuno-labeled RPS-producing *streptococci *(red).

Laser confocal microscopy performed in conjunction with different specific antibodies has been used to examine the distribution of RPS-bearing streptococci and type 2 fimbriated *A. naeslundii *in early biofilms formed in vivo on retrievable enamel chips [21]. Bacteria labeled for cell surface RPS were seen in large numbers on chips following 8 hr incubation in vivo. Other bacteria labeled with antibodies specific for strains of *A. naeslundii *were also present, but in much lower numbers. Significantly, the latter cells (*i.e*., type 2 fimbriated *A. naeslundii*) were found almost exclusively within microcolonies of RPS-producing bacteria, thereby supporting the role of RPS-mediated interbacterial adhesion in early biofilm formation. Moreover, *A. naeslundii *has been identified within spreading monolayers of RPS-producing streptococci formed during early in vivo colonization of the enamel surface (Fig. 3).

**Figure 4:**
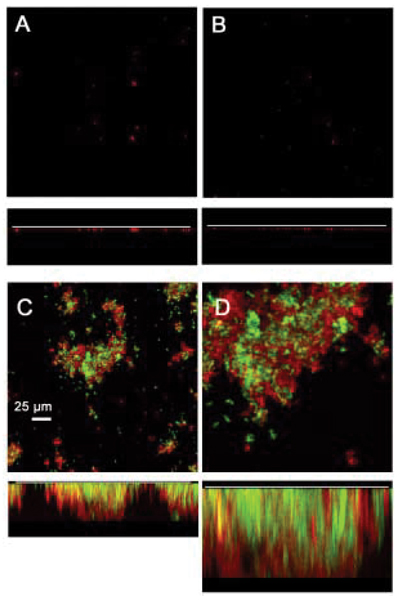
RPS-mediated interbacterial adhesion promotes the growth of *S. oralis *34 and *A. naeslundii *T14V as a biofilm in flowing human saliva. Flow cells were incubated overnight at 37C following inoculation with different combinations of wild type strain 34 or an RPS-negative mutant and wild type strain T14V or a type 2 fimbriae deficient mutant. Biofilms were labeled with conjugated antibodies to detect *streptococci *(red) and *actinomyces *(blue) and examined confocal fluorescent microscopy.

The ecological role of RPS-mediated interbacterial adhesion may be to promote the formation of food chains and other mutualistic interactions that require close contact between different bacteria. Evidence for this possibility has been obtained using a flowcell model of biofilm formation in which human saliva is the sole source of nutrients for bacterial growth [22]. Biofilm formation was limited or did not occur under these conditions when either RPS-producing *S. oralis *34 or type 2 fimbriated *A. naeslundii *T14V were introduced separately into flowcells. However, a luxuriant mixed-species biofilm formed when these two strains were introduced together. These observations have recently been extended by the results of similar experiments performed with these wild-type strains and corresponding mutant strains that specifically lack cell-surface RPS or type 2 fimbriae. As expected, wild-type *S. oralis *34 and *A. naeslundii *T14V formed a luxuriant biofilm (Fig. 4). However, biofilm formation was greatly reduced when either wild-type strain was paired with the non-adherent mutant of the other cell type (*i.e*., RPS-negative *S. oralis *34 or type 2 fimbriae-negative *A. naeslundii *T14V). Thus, RPS-mediated interbacterial adhesion appears to promote the establishment of a mutualistic association between *S. oralis *and *A. naeslundii *that is essential for luxuriant growth and mixed-species biofilm formation in flowing human saliva.

### Characterization of RPS-Producing Bacteria from Different Individuals

The identification of six structural types of RPS is based on the characterization of representative strains of the four RPS-producing species. Consequently, it is unclear how many different RPS-producing strains occur within the streptococcal flora of any one individual. To address this question, we have begun to characterize these bacteria from individual volunteers. This involves the identification of RPS-producing clones from samples of 8 hr biofilms by colony immunoblotting with a cocktail of RPS-specific antibodies and the subsequent characterization of individual clones by the reactions with specific antibodies and adhesins. Initial results of these studies indicate the production of one predominant type of RPS by the resident streptococcal flora of each individual examined to date. Thus, 22 RPS-producing isolates from one volunteer all produced type 1Gn RPS while 19 RPS-producing isolates from another individual all produced type 2Gn RPS. In addition, 21 RPS-producing isolates from a third individual included 19 that produced type 2Gn RPS and two that produced type 1Gn RPS. PCR-based genotyping [23] of these isolates suggests the presence of from one to three different RPS-producing clones per individual. These clones appear to be *S. oralis *or *S. gordonii *based on sequencing of a housekeeping gene [24]. Whether RPS-producing clones of different individuals are subject to replacement or to chromosomal rearrangement over time as has been described for strains of *S. mitis *[25] and *S. oralis *[26] remains to be determined.

## Conclusion

Over 90% of the RPS-producing *streptococci *identified from samples of early supragingival plaque from each of five individuals are type 1Gn or 2Gn RPS producing clones. The preponderance of these bacteria in the plaque samples examined to date raises a number of intriguing possibilities concerning the occurrence and distribution of the *streptococci *that produce other structural types. Clearly, one possibility is that the streptococcal floras of certain individuals will be found to produce type 2G, 3G, 4Gn or 5Gn RPS, thereby indicating significant differences in RPS production between individuals. Alternatively, the RPS-producing flora of each individual, including those that have been examined, may be more complex than is presently recognized. If so, *streptococci *that produce structural types other than type 1Gn or 2Gn RPS may emerge as the early biofilm community matures or perhaps appear during the colonization of other oral sites. In any case, it will be important to determine whether RPS-producing *streptococci*, especially those that produce Gn- and G-types, exist within distinct biofilm communities. The existence of RPS-based differences in the composition of biofilm communities would firmly establish the recognition role of these polysaccharides in biofilm development and could provide important new insights into the etiology and pathogenesis of biofilm-related oral diseases, including dental caries.

Another intriguing area of investigation involves the natural history of RPS-producing *streptococci *in the human host. Available findings indicate that these bacteria are transmitted from adults to infants shortly after tooth eruption. It is unclear, however, whether the resident RPS-producing streptococcal flora, once acquired, is stable, or whether changes in the population of RPS-producing clones occur over the lifetime of each individual. New structural types of RPS may evolve gradually and be disseminated by transmission between individuals. Alternatively, new types may arise frequently by the horizontal transfer of genes for RPS biosynthesis between different streptococcal strains and species within the resident flora of each individual. Current molecular studies of RPS production are directed toward distinguishing these possibilities.

## Competing interests

The authors declare that they have no competing interests.

## Authors' contributions

All authors read and approved the final manuscript.
